# 3D-MRI rendering of the anatomical structures related to acupuncture points of the Dai mai, Yin qiao mai and Yang qiao mai meridians within the context of the WOMED concept of lateral tension: implications for musculoskeletal disease

**DOI:** 10.1186/1471-2474-8-33

**Published:** 2007-04-10

**Authors:** Roy Moncayo, Ansgar Rudisch, Christian Kremser, Helga Moncayo

**Affiliations:** 1 WOMED, Karl-Kapferer-Strasse 5, A-6020 Innsbruck, Austria; 2 Innsbruck Medical University, Department of Radiology I, A-6020 Innsbruck, Austria

## Abstract

**Background:**

A conceptual model of lateral muscular tension in patients presenting thyroid associated ophthalmopathy (TAO) has been recently described. Clinical improvement has been achieved by using acupuncture on points belonging to the so-called extraordinary meridians. The aim of this study was to characterize the anatomical structures related to these acupuncture points by means of 3D MRI image rendering relying on external markers.

**Methods:**

The investigation was carried out the index case patient of the lateral tension model. A licensed medical acupuncture practitioner located the following acupuncture points: 1) Yin qiao mai meridian (medial ankle): Kidney 3, Kidney 6, the plantar Kidney 6 (Nan jing description); 2) Yang qiao mai meridian (lateral ankle): Bladder 62, Bladder 59, Bladder 61, and the plantar Bladder 62 (Nan jing description); 3) Dai mai meridian (wait): Liver 13, Gall bladder 26, Gall bladder 27, Gall bladder 28, and Gall bladder 29. The points were marked by taping a nitro-glycerin capsule on the skin. Imaging was done on a Siemens Magnetom Avanto MR scanner using an array head and body coil. Mainly T1-weighted imaging sequences, as routinely used for patient exams, were used to obtain multi-slice images. The image data were rendered in 3D modus using dedicated software (Leonardo, Siemens).

**Results:**

Points of the Dai mai meridian – at the level of the waist – corresponded to the obliquus externus abdominis and the obliquus internus abdominis. Points of the Yin qiao mai meridian – at the medial side of the ankle – corresponded to tendinous structures of the flexor digitorum longus as well as to muscular structures of the abductor hallucis on the foot sole. Points of the Yang qiao mai meridian – at the lateral side of the ankle – corresponded to tendinous structures of the peroneus brevis, the peroneous longus, and the lateral surface of the calcaneus and close to the foot sole to the abductor digiti minimi.

**Conclusion:**

This non-invasive MRI investigation has revealed the anatomical relations of acupuncture points belonging to 3 of the so-called extraordinary meridians. We conclude that the clinically developed "WOMED concept of lateral tension" is related to tendino-muscular structures.

## Background

Acupuncture is a fundamental component of Traditional Chinese Medicine (TCM) used in therapeutical settings. Textbooks on acupuncture describe a learning process based on the observation of changes of the surface of the body in relation to disease conditions. Based on these clinical experiences, the so-called meridians have been classically recognized and described [1]. The naming of the acupuncture points reflects the knowledge of the universe, i.e. the structures found in Heaven and Earth, of early Chinese medicine such as described in the Nan jing textbook [2,3].

Even though acupuncture points have classical descriptions related to location, interactions and effects, the mechanisms by which acupuncture works are not fully known. Based on the pioneering work of Rasmussen and Penfield, who showed the correspondence between skin and cortical representation [4], central effects elicited after acupuncture needling can be characterized in discrete brain areas [5-8] as well as at the spinal level [9,10] using fMRI. Few investigations have tried to characterize the anatomical basis of acupuncture points [11-14]. Langevin and Yandow have described an intramuscular connective tissue cleavage plane at an acupuncture point based on ultrasound examination with a 7 MHz probe [14]. Besides these attempts, little has been done to implement high resolution imaging technology for the description of the anatomy of acupuncture points in-vivo.

**Figure 1:**
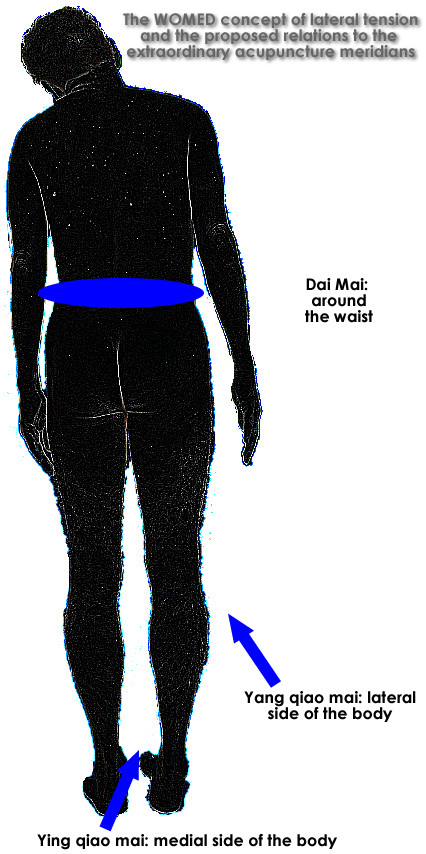
Schematic representation of the "WOMED concept of lateral tension". The body is found in an arch-like position which includes foot inversion, eccentric muscle position of the shank, calf and hip, as well as head falling to the side (based on [15]).

Based on clinical observations gained from patients with thyroid associated ophthalmopathy (TAO) we have recently developed the a biomechanical model related to low-grade inflammation of the connective tissue [15]. In this model the lateral aspect of the body is found to be in an arch-like position. This position can affect the tension of the shanks producing a feeling of fullness or tightness. In addition, the foot of the affected side is in inversion and the head falls to the contra lateral side. Taut bands can be found on the lateral abdominal muscles. These postural alterations were amenable to correction by means of acupuncture based on points that belong to the so-called extraordinary meridians. The extraordinary meridians in question, the Yang qiao mai and the Yin qiao mai, relate areas of the body from the foot to the eye (Figure 1). The Yang qiao mai is described as ascending from the heel to the lateral malleolus, to the lateral shank, to the lateral abdomen and ending on the lateral corner of the eyes. The Yin qiao mai ascends in a parallel fashion from the heel to the medial malleolus ending on a point located on the medial corner of the eye. The Dai mai is located at the level of the waist.

Following an initial study which used gold acupuncture needles for direct MRI imaging [16] we have now relied on the use of external markers to describe the anatomy related to acupuncture points on MRI data sets. The external marker technique has been used by us since several years [17] and is based on the imaging ability of fluids under T1-weighted MRI conditions. Dedicated rendering software has been used for the 3D anatomical analysis of the data sets.

## Methods

### Study subject

The patient is a 51 year-old male who had presented pain on the lateral sides of the right shank 1 year ago. His previous medical history revealed an episode of sudden foot inversion during eccentric muscle exercise (downhill running) which had happened 10 years ago. Two years after the traumatic event pain in the foot appeared. Both a bone scintigraphy as well as an X-ray examination was uneventful. Due to the pain, regular athletic training had been reduced in intensity in the following 5 years. Regular training for triathlon was started again three years ago; however pain reappeared on the right leg affecting the lateral aspect of the shank. On examination the skin felt warm and tense and the range of motion of the right ankle was reduced. Anti-inflammatory medication and physical medicine methods attained only temporary help. Acupuncture treatments based on meridian concepts, i.e. treatment according to the topographical distribution of acupuncture meridians, were partially successful. In spite of these therapeutic procedures, complaints re-appeared after some months. Finally the acupuncture treatment strategy was changed in order to include concepts of the so-called extraordinary meridians. Therapy consisted of needling of the Bl62 point (Shen mai) and of the Gb26 point (Dai mai) together with blood letting at the distal point of the bladder meridian, Bl67. The treatment was repeated three times after which the symptoms subsided. The skin temperature on the shank became normal and the feeling of lateral tension disappeared. The present MRI study was conducted as a control procedure. Institutional ethical approval was obtained for the study. The individual gave his consent to participate in the study.

### Localization of the acupuncture points

A licensed medical acupuncture practitioner (RM) localized the points in question following classical descriptions. In order to avoid magnetic interferences with the imaging equipment, external markers were taped onto each point. External fiducial markers can be used successfully for image fusion studies [17-19]. The acupuncture points chosen for the investigation belong to the Dai mai (or the girdling vessel), the Yin qiao mai (or yin motility vessel) and the Yang qiao mai (or Yang motility vessel). These meridians correspond roughly to the level of the waist, and to the medial and lateral part of the lower limb, trunk and head, respectively. The classical characteristics of the points can be found in [20]: "The Dai mai point is located in the depression one inch and eight fen below the region of the free ribs (11^th ^rib). This point corresponds to the intersection jiaohui point of the foot shao yang gall bladder channel and the girdling vessel. The Shen mai point is located in the depression five fen below the outer anklebone. It corresponds to the confluence-jiaohui point of the eight extraordinary vessels (yang motility vessel)". The inclusion of the plantar level of both Kidney 6 and Urinary Bladder 62 was done based on the ancestral description of these points as can be found in the Nan jing, Chapter 28 where it can be read that these points rise from the heel. Physiologically, these areas are known to deliver important information for muscle activation involved in foot and lower limb movements [21,22].

It should be noted that the location of acupuncture points is based on a proportional measure system where the unit is the cun. One cun is defined either as "the distance between the ends of the creases of the interphalangeal joints of the middle finger at their widest point" or "the width of the interphalangeal joint of the thumb" [1]. One tenth of a cun is one fen. Metric units are not applicable in acupuncture.

### MRI imaging

MRI imaging was done on a Siemens Magnetom Avanto MR scanner using a head array and a body coil. 3D image sets were obtained from each region studied. Imaging of the abdominal region was done as follows: body array coil, fat-saturated T1-weighted 3D gradient-echo sequence for MRI of the body (volumetric interpolated breath-hold examination, VIBE) [23]: TR = 4.36 ms, TE = 2.22 ms, flip angle: 10°, coronal orientation, FOV: 400 mm with rectangular configuration (87.5%) in phase-encoding dimension, number of slices: 72, slice resolution: 63%, 6/8 slice partial Fourier, slice thickness: 3 mm, base resolution: 256, phase resolution: 65%, 7/8 phase partial Fourier, band with: 350 Hz per Px, parallel imaging mode: GRAPPA, acceleration factor: 2, total acquisition time: 15 s. Imaging of the foot region was done using a matrix head coil.

Nitro-glycerin capsules were used as external fiducial markers for the location of the acupuncture points. In previous publications we have demonstrated the utility of these capsules in MRI studies [17]. Under T1-weighted conditions, which are ideal for anatomical studies, the fluid content of the capsules has a short T1-relaxation time which results in a bright signal. Since the capsules are on the surface of the body no interferences with underlying structures can be expected.

### MRI image processing

The image data sets were rendered in 3D modus using dedicated software (Leonardo, Siemens, Erlangen, Germany). Three levels of image peeling, i.e. surface to tendons and ligament levels were generated. This allowed us to look at the underlying structures. The images were analyzed by an experienced musculoskeletal radiologist specialized in MRI (AR). The anatomical structures were sought at the level that would correspond to the tip of an inserted acupuncture needle.

## Results

The routine evaluation of the MRI images did not reveal any pathological changes in the subject studied. The description of the location of the acupuncture points and the anatomical structures being recognized are summarized in Table 1.

Points of the Dai mai meridian corresponded mostly to superficial muscular structures of the abdomen, i.e. the obliquus externus abdominis and the obliquus internus abdominis. Points of the Yin qiao mai meridian at the level of the ankle corresponded to tendinous structures of the flexor digitorum longus as well as muscular structures of the abductor hallucis on the foot sole. Points of the Yang qiao mai meridian at the level of the ankle corresponded to tendinous structures of the peroneous brevis, the peroneous longus, the lateral surface of the calcaneus and on the foot sole to the abductor digiti minimi.

## Discussion

In a short historical analysis of acupuncture Fee et al. [24] have stated that: "The traditional Chinese system of meridians does not correspond with any anatomical structures recognized by Western medicine". This statement is valid since until 2002, when Fee et al. published their article [24], no attempts had been undertaken to analyze in-vivo acupuncture meridians. We are aware of 4 studies that relied on anatomical slices to address this question [11-14], however no high resolution imaging methods were used in-vivo. By means of 3D rendering of MRI data we have been able to demonstrate the relation of acupuncture points to musculo-skeletal structures of the body. In the following sections we will discuss the physiological importance of these findings.

The MRI study was conducted using external fiducial markers which are otherwise needed for image fusion in different imaging modalities (e.g. MRI + PET or MRI + SPECT or MRI + CT + PET + SPECT) [18,19,25-30]. Due to the fluid and oily characteristics of nitro-glycerin capsules, a bright signal in T1-weighted sequences will be produced. Finally, since the capsules are placed on the surface of the body, no interactions with the underlying anatomy will be produced. This approach has not been used in this context before.

Our recent description of the WOMED concept of lateral tension as a mechanism related to low level inflammation of the connective tissue in patients with thyroid associated ophthalmopathy (TAO) included the diagnostic-therapeutic use of specific acupuncture points that belong to the so-called extraordinary meridians [15]. Based on our clinical experience we can include other groups of patients with muscular affections that fit into this model, e.g. ankle joint instability, myalgia, fibromyalgia, and low back pain. Furthermore, the model might be of relevance for patients with moving toes [31], since this feature was also found in TAO patients [15]. Acupressure on points of the extra-ordinary meridians described here was able to regulate this inducible phenomenon of moving toes.

Figure 1 depicts the general appearance of the lateral tension model which includes changes in the ankle, the shank, the trunk, the neck, the head and the eyes. Within the conceptual frame of the extraordinary meridians several of these structures can be considered to constitute a functional unit. The lateral side of the ankle and leg as well as of the trunk, the neck and head correspond to the Yang qiao mai or Yang motility vessel; the medial side corresponds to the Ying qiao mai or Yin motility vessel; the structures of the trunk at the level of the waist correspond to the Dai mai or girdling vessel [32-35]. This integrative concept allows clinicians to recognize pathophysiological changes that might be distant to the site where local changes appear, e.g. eye motility changes that are related to foot inversion in TAO patients.

Besides the anatomical correlates discussed above, we would like to mention some relevant biomechanical data. Anticipation of limb movement involves the activation of the trunk and abdominal muscles [36]. This activation can be initiated either by arm or leg movements. It follows, that the abdominal muscles have to react constantly to movement-induced activation. The posterior layer of the thoracolumbar fascia exerts a function in load transfer between the spine and the legs [37-40]. Urquhart et al. have described the anatomical characteristics of the transversus abdominis, the obliquus internus, and externus abdominis muscles in relation to limb movements [41,42]. It is interesting to note, that the middle region of the abdominal wall in their study, corresponds to the location of the Dai mai point (Gb26). Their localization was described by them as: "at the level of the 11^th ^costal cartilage, halfway between the iliac crest and the rib cage..." [41]. Furthermore the orientation of the obliquus internus corresponds to the trajectory that can be traced between the points Gb27 and Gb 28. These muscles also influence compression of the sacroiliac joint [43], thus suggesting a relation to clinical conditions of low back pain [44]. Altered function of the trunk muscles can indeed occur in cases of LBP [45]. In addition, contraction of the abdominal muscles can result in the production of a band-like change [46]. We have found such "taut-bands" on the lateral abdominal wall in the series of patients with TAO [15]. Evidence showing metabolic activation of the lateral abdominal muscles have been mentioned in the description of the model of lateral tension [15]. This phenomenon appears especially around the Dai mai or Gall bladder 26 point. Acupuncture treatment at this level resolves its tightness. Besides these muscular aspects, several publications have described the determinant role of fascial structures in several diseases [38,47-53]. In summary, tendino-muscular structures seem to relevant in transmitting force and coordinating movements. Taut bands can be viewed as interfering structures that lead to increased tension on the body. Taut bands can appear when eccentric muscle action is present [15,54].

## Conclusion

Surface rendering procedures of MRI data sets together with external fiducial markers can be used successfully to describe the in-vivo anatomical relations of acupuncture points. Our data suggest a close relation of acupuncture points of the Yang and Yin motility vessels as well as of the Dai mai to tendino-muscular structures. Biomechanical data point out their importance in posture and locomotion. New examination and therapy procedures based on the "WOMED concept of lateral tension" might be of benefit in clinical practice.

## Competing interests

The author(s) declare that they have no competing interests.

## Authors' contributions

All authors contributed equally to this work: HM and RM have developed the concept of lateral tension, RM did the acupuncture work. AR, an experienced musculoskeletal radiologist, did the image interpretation. CK an experienced radiology physicist made and processed the MRI studies.

## Pre-publication history

The pre-publication history for this paper can be accessed here:



## Figures and Tables

**Figure 2:**
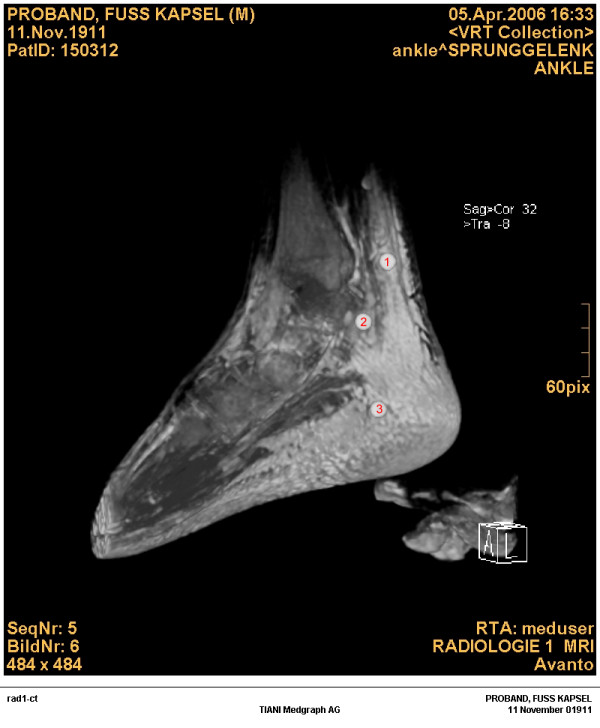
3-D volume rendering of the medial portion of the foot (Yin qiao mai meridian): the points studied correspond to: 1) Tendino-muscular segment of the flexor digitorum longus, 2) Tendon of the flexor digitorum longus on the talus, and 3) abductor hallucis muscle.

**Figure 3:**
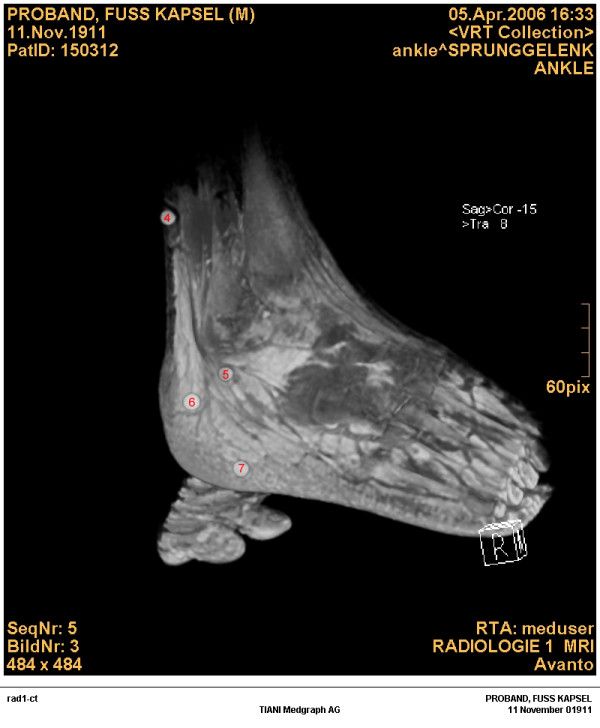
3-D volume rendering of the lateral portion of the foot (Yang qiao mai meridian): the points studied correspond to: 4) Tendino-muscular segment of the peroneus brevis, 5) Tendon of the peroneus longus on the lateral ankle, 6) Lateral surface of the calcaneus, 7) abductor digiti minimi muscle.

**Figure 4:**
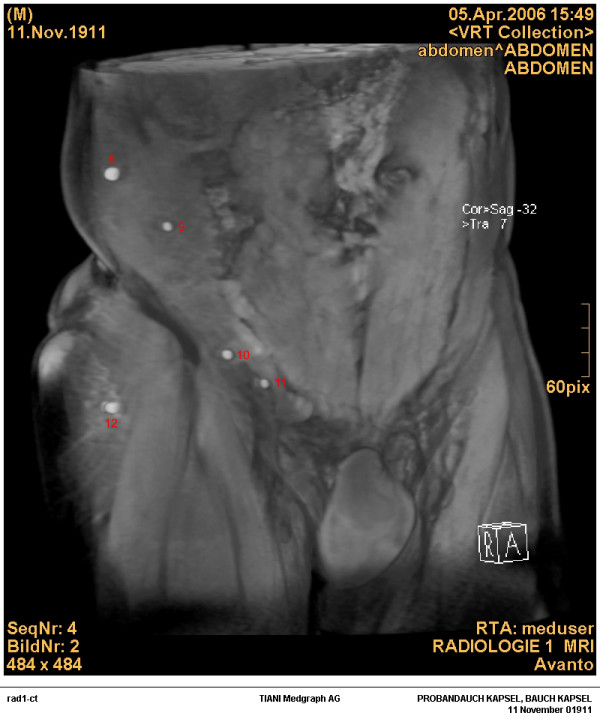
3-D volume rendering of the lateral abdomen (Dai mai meridian): the points studied correspond to: 8) obliquus externus abdominis muscle, 9) obliquus externus abdominis muscle, 10) obliquus internus abdominis muscle, 11) obliquus internus abdominis muscle, 12) glutaeus medius muscle.

**Table 1:** 

**Number**	**Location**	**Acupuncture point**	**Anatomic structure**
1	Half way between the medial malleolus and the achilles tendon	Kidney 3	Tendino-muscular segment of the flexor digitorum longus
2	Depression directly inferior to the medial	Kidney 6	Tendon of the flexor digitorum longus on the talus
3	From the heel rising to the medial malleolus	Kidney 6 plantar	abductor hallucis muscle
4	On the lateral ankle, in the depression midway between the external malleolus and the tendon calcaneus	Bladder 60	Tendino-muscular segment of the peroneus brevis
5	Depression directly inferior to the lateral malleolus	Bladder 62	Tendon of the peroneus longus on the lateral ankle
6	Inferior and posterior to the lateral malleolus	Baldder 61	Lateral surface of the calcaneus
7	From the heel rising to the lateral malleolus	Bladder 62 plantar	abductor digiti minimi muscle
8	Slightly inferior and anterior to the tip of the 11th rib	Liver 13	obliquus externus abdominis muscle
9	Mid point between the 11th rib and the iliac crest	Gall bladder 26	obliquus externus abdominis muscle
10	Medial and inferior to the ASIS	Gall bladder 27	obliquus internus abdominis muscle
11	App. 1 cm medial and inferior to Gall bladder 27	Gall bladder 28	obliquus internus abdominis muscle
12	Mid point between the greater trochanter and the ASIS	Gall bladder 29	glutaeus medius muscle

## References

[B1] Deadman P, Al-Khafaji M, Baker K (2001). A manual of acupuncture.

[B2] Lo V (2003). The territory between life and death. Essay review. Med Hist.

[B3] Shi C (2002). [Review on fragmentary volume of original block - printed edition of Nan jing ben yi (Gist of the Classic of Questioning)]. Zhonghua Yi Shi Za Zhi.

[B4] Rasmussen T, Penfield W (1947). Further studies of the sensory and motor cerebral cortex of man. Fed Proc.

[B5] Campbell A (2006). Point specificity of acupuncture in the light of recent clinical and imaging studies. Acupunct Med.

[B6] Li K, Shan B, Xu J, Liu H, Wang W, Zhi L, Li K, Yan B, Tang X (2006). Changes in FMRI in the human brain related to different durations of manual acupuncture needling. J Altern Complement Med.

[B7] Nakagoshi A, Fukunaga M, Umeda M, Mori Y, Higuchi T, Tanaka C (2005). Somatotopic representation of acupoints in human primary somatosensory cortex: an FMRI study. Magn Reson Med Sci.

[B8] Shen J (2001). Research on the neurophysiological mechanisms of acupuncture: review of selected studies and methodological issues. J Altern Complement Med.

[B9] Guo D, Guan X, Wang C (1996). [Segmental influence of dorsal root action potentials evoked by stimulating the acupoints after acupuncture along meridians]. Zhen Ci Yan Jiu.

[B10] Li G, Ng MC, Wong KK, Luk KD, Yang ES (2005). Spinal effects of acupuncture stimulation assessed by proton density-weighted functional magnetic resonance imaging at 0.2 T. Magn Reson Imaging.

[B11] Peuker E, Cummings M (2003). Anatomy for the acupuncturist--facts & fiction. 1: The head and neck region. Acupunct Med.

[B12] Peuker E, Cummings M (2003). Anatomy for the acupuncturist--facts & fiction 2: The chest, abdomen, and back. Acupunct Med.

[B13] Peuker E, Cummings M (2003). Anatomy for the acupuncturist--facts & fiction. 3: Upper & lower extremity. Acupunct Med.

[B14] Langevin HM, Yandow JA (2002). Relationship of acupuncture points and meridians to connective tissue planes. Anat Rec.

[B15] Moncayo R, Moncayo H (2007). A musculoskeletal model of low grade connective tissue inflammation in patients with thyroid associated ophthalmopathy (TAO): the WOMED concept of lateral tension and its general implications in disease. BMC Musculoskelet Disord.

[B16] Moncayo R, Rudisch A, Diemling M, Kremser C (2007). In-vivo visualisation of the anatomical structures related to the acupuncture points Dai mai and Shen mai by MRI: A single-case pilot study. BMC Med Imaging.

[B17] Sweeney RA, Bale RJ, Moncayo R, Seydl K, Trieb T, Eisner W, Burtscher J, Donnemiller E, Stockhammer G, Lukas P (2003). Multimodality cranial image fusion using external markers applied via a vacuum mouthpiece and a case report. Strahlenther Onkol.

[B18] Kainz H, Bale R, Donnemiller E, Gabriel M, Kovacs P, Decristoforo C, Moncayo R (2003). Image fusion analysis of (99m)Tc-HYNIC-octreotide scintigraphy and CT/MRI in patients with thyroid-associated orbitopathy: the importance of the lacrimal gland. Eur J Nucl Med Mol Imaging.

[B19] Profanter C, Wetscher GJ, Gabriel M, Sauper T, Rieger M, Kovacs P, Bale R, Prommegger R (2004). CT-MIBI image fusion: a new preoperative localization technique for primary, recurrent, and persistent hyperparathyroidism. Surgery.

[B20] Ellis A, Wiseman N, Boss K (1989). Grasping the wind.

[B21] Andersen OK, Sonnenborg FA, Arendt-Nielsen L (2001). Reflex receptive fields for human withdrawal reflexes elicited by non-painful and painful electrical stimulation of the foot sole. Clin Neurophysiol.

[B22] Andersen OK, Sonnenborg FA, Arendt-Nielsen L (1999). Modular organization of human leg withdrawal reflexes elicited by electrical stimulation of the foot sole. Muscle Nerve.

[B23] Rofsky NM, Lee VS, Laub G, Pollack MA, Krinsky GA, Thomasson D, Ambrosino MM, Weinreb JC (1999). Abdominal MR imaging with a volumetric interpolated breath-hold examination. Radiology.

[B24] Fee E, Brown TM, Lazarus J, Theerman P (2002). Exploring acupuncture: ancient ideas, modern techniques. Am J Public Health.

[B25] Sweeney RA, Bale R, Auberger T, Vogele M, Foerster S, Nevinny-Stickel M, Lukas P (2001). A simple and non-invasive vacuum mouthpiece-based head fixation system for high precision radiotherapy. Strahlenther Onkol.

[B26] Profanter C, Prommegger R, Gabriel M, Moncayo R, Wetscher GJ, Lang T, Bale R (2003). Computed axial tomography-MIBI image fusion for preoperative localization in primary hyperparathyroidism. Am J Surg.

[B27] Prommegger R, Bale R, Ensinger C, Sauper T, Profanter C, Knoflach M, Moncayo R (2003). Gastric carcinoid type I tumour: new diagnostic and therapeutic method. Eur J Gastroenterol Hepatol.

[B28] Gabriel M, Hausler F, Bale R, Moncayo R, Decristoforo C, Kovacs P, Virgolini I (2005). Image fusion analysis of (99m)Tc-HYNIC-Tyr(3)-octreotide SPECT and diagnostic CT using an immobilisation device with external markers in patients with endocrine tumours. Eur J Nucl Med Mol Imaging.

[B29] Profanter C, Prommegger R, Moncayo R, Bale R (2005). CT-MIBI image fusion. Wien Klin Wochenschr.

[B30] Weiss H, Kafka-Ritsch R, Zitt M, Klaus A, Heute D, Moncayo R, Kovacs P, Bale R, Öfner D (2005). The Innsbruck sentinel lymph node study in colorectal cancer - A pilot study. Eur Surg.

[B31] Dressler D, Thompson PD, Gledhill RF, Marsden CD (1994). The syndrome of painful legs and moving toes. Mov Disord.

[B32] Maciocia G (2006). The Channels of Acupuncture: Clinical Use of the Secondary Channels and Eight Extraordinary Vessels.

[B33] Ross J (1995). Acupuncture Point Combinations: The Key to Clinical Success.

[B34] Maciocia G (2004). Diagnosis in Chinese Medicine A comprehensive guide.

[B35] Kirschbaum B (2000). Die 8 außerordentlichen Gefäße in der traditionellen chinesischen Medizin.

[B36] Hodges PW, Richardson CA (1996). Inefficient muscular stabilization of the lumbar spine associated with low back pain. A motor control evaluation of transversus abdominis. Spine.

[B37] Vleeming A, Pool-Goudzwaard AL, Stoeckart R, van Wingerden JP, Snijders CJ (1995). The posterior layer of the thoracolumbar fascia. Its function in load transfer from spine to legs. Spine.

[B38] Barker PJ, Briggs CA (1999). Attachments of the posterior layer of lumbar fascia. Spine.

[B39] Barker PJ, Briggs CA, Bogeski G (2004). Tensile transmission across the lumbar fasciae in unembalmed cadavers: effects of tension to various muscular attachments. Spine.

[B40] Barker PJ, Guggenheimer KT, Grkovic I, Briggs CA, Jones DC, Thomas CD, Hodges PW (2006). Effects of tensioning the lumbar fasciae on segmental stiffness during flexion and extension: Young Investigator Award winner. Spine.

[B41] Urquhart DM, Barker PJ, Hodges PW, Story IH, Briggs CA (2005). Regional morphology of the transversus abdominis and obliquus internus and externus abdominis muscles. Clin Biomech (Bristol, Avon).

[B42] Urquhart DM, Hodges PW (2005). Differential activity of regions of transversus abdominis during trunk rotation. Eur Spine J.

[B43] Richardson CA, Snijders CJ, Hides JA, Damen L, Pas MS, Storm J (2002). The relation between the transversus abdominis muscles, sacroiliac joint mechanics, and low back pain. Spine.

[B44] Renkawitz T, Boluki D, Grifka J (2006). The association of low back pain, neuromuscular imbalance, and trunk extension strength in athletes. Spine J.

[B45] Lariviere C, Gagnon D, Loisel P (2000). The comparison of trunk muscles EMG activation between subjects with and without chronic low back pain during flexion-extension and lateral bending tasks. J Electromyogr Kinesiol.

[B46] Hides J, Wilson S, Stanton W, McMahon S, Keto H, McMahon K, Bryant M, Richardson C (2006). An MRI investigation into the function of the transversus abdominis muscle during "drawing-in" of the abdominal wall. Spine.

[B47] Aquino A, Payne C (1999). Function of the plantar fascia. Foot.

[B48] Huijing P (1999). Muscular force transmission: a unified, dual or multiple system? A review and some explorative experimental results. Arch Physiol Biochem.

[B49] Maas H, Baan GC, Huijing PA (2001). Intermuscular interaction via myofascial force transmission: effects of tibialis anterior and extensor hallucis longus length on force transmission from rat extensor digitorum longus muscle. J Biomech.

[B50] Robertson S (2001). Integrating the fascial system into contemporary concepts on movement dysfunction. J Man Manipul Ther.

[B51] Theodorou DJ, Theodorou SJ, Resnick D (2002). MR imaging of abnormalities of the plantar fascia. Semin Musculoskelet Radiol.

[B52] Loukas M, Louis J, Van der Wall B, Hallner B, Tucker JJ, Esguerra F, Colborn GL (2006). Iliolumbar membrane, a newly recognised structure in the back. Folia Morphologica.

[B53] Stecco C, Porzionato A, Macchi V, Tiengo C, Parenti A, Aldegheri R, Delmas V, De Caro R (2006). Histological characteristics of the deep fascia of the upper limb. Ital J Anat Embryol.

[B54] Itoh K, Okada K, Kawakita K (2004). A proposed experimental model of myofascial trigger points in human muscle after slow eccentric exercise. Acupunct Med.

